# Chronic Cerebral Ischaemia Forms New Cholinergic Mechanisms of Learning and Memory

**DOI:** 10.4061/2010/954589

**Published:** 2010-12-20

**Authors:** E. I. Zakharova, Z. I. Storozheva, A. M. Dudchenko, A. A. Kubatiev

**Affiliations:** ^1^Institute of General Pathology and Pathophysiology, RAMS, Baltic street 8, Moscow 125315, Russia; ^2^P.K. Anokhin' Institute of Normal Physiology, RAMS, Baltic street 8, Moscow 125315, Russia

## Abstract

The purpose of this research was a comparative analysis of cholinergic synaptic organization following learning and memory in normal and chronic cerebral ischaemic rats in the Morris water maze model. Choline acetyltransferase and protein content were determined in subpopulations of presynapses of “light” and “heavy” synaptosomal fractions of the cortex and the hippocampus, and the cholinergic projective and intrinsic systems of the brain structures were taken into consideration. We found a strong involvement of cholinergic systems, both projective and intrinsic, in all forms of cognition. Each form of cognition had an individual cholinergic molecular profile and the cholinergic synaptic compositions in the ischaemic rat brains differed significantly from normal ones. Our data demonstrated that under ischaemic conditions, instead of damaged connections new key synaptic relationships, which were stable against pathological influences and able to restore damaged cognitive functions, arose. The plasticity of neurochemical links in the individual organization of certain types of cognition gave a new input into brain pathology and can be used in the future for alternative corrections of vascular and other degenerative dementias.

## 1. Introduction

Vascular dementia sometimes can precede or accompany Alzheimer's disease, and in these cases the development of Alzheimer's disease becomes more dramatic. The dementia of ischaemic type and Alzheimer's disease is synergistic or additive in the earliest stages of Alzheimer's disease, although the interactive mechanisms are not known [1, 2]. Both types of dementia are neurodegenerative diseases and for both the dysfunction and degeneration of cholinergic projective systems in the cortex and the hippocampus from the forebrain nuclei are critical [3–8]. A number of studies on animal models demonstrated a possible trigger role of cholinergic projective neurons in brain ischaemia. The early activation of cholinergic projective neurons was found to occur simultaneously with glutamatergic activation in the cortex and the hippocampus [9–12]. Correlations between the development of cholinergic dysfunctions and the destruction of pyramidal neurons in the hippocampus [6, 13, 14], and also damage to the cognitive functions of animals [4–8, 10], led to the presumption that a dysfunction in cholinergic afferents plays a major role in the development of ischaemic pathologies [9, 12, 14, 15]. 

 Modern electrophysiology accumulated numerous data that interneurons of the cortex and the hippocampus actively participate in the modulation of neuronal activity including the hippocampal pyramidal neurons [16–18]. It was revealed that the cholinergic effects on the interneurons of the cortex and the hippocampus was substantially mediated through nicotinic receptors (nAChRs) [16–21]. On the other hand the role of the cholinergic interneurons in behavioural, and neurodegenerative mechanisms is still unknown.

Our investigations on the “light” and “heavy” synaptosomal fractions of the cortex and the hippocampus allowed the study of the major cholinergic projection systems of the cortex and the hippocampus and their minor intrinsic systems of cholinergic interneurons. According to immunochemical data, both the cortex and the hippocampus have two basic sources of cholinergic innervations. The first major sources are the neuronal projections from the forebrain nuclei basalis magnocellularis into the cortex (precursor of the Meynert nucleus in primates and humans) and projections from the forebrain medial septal nuclei and vertical limb nuclei of the diagonal band of Broca into the hippocampus. The second minor source is the interneurons (intrinsic neurons) [22–28]. The synaptosomes are presynaptic parts of synapses with their junction complexes; these shall be termed presynapses in the present study. We previously showed that for both the cortex and the hippocampus the cholinergic presynapses from different sources are isolated in different synaptosomal fractions during preparation in the sucrose density gradient. The presynapses of cholinergic projections from the forebrain nuclei are accumulated mainly in the light synaptosomal fractions whereas the presynapses of cholinergic interneurons are accumulated mainly in the heavy synaptosomal fractions [29–31]. It is probable that the heavy synaptosomal fraction of the hippocampus may also accumulate a small part of the cholinergic projective presynapses (lateral projection pathway into the hippocampus [32]) [31]. Our studies on the cortical synaptosomal fractions of cats allowed suggest the involvement of the cholinergic interneurons in cognitive functions [29]. In the studies on the cortical and hippocampal synaptosomal fractions of rat we revealed that during the first three hours of chronic brain ischaemia the cholinergic projective neurons were reactive, as were the interneurons of the cortex and the hippocampus as well [33].

At present, the molecular, genetic and neurochemical mechanisms of cognitive functions are widely investigated in different behavioural models and widely discussed as well. Some among these data induce to revise generally conception that memory formation involves an irreversible passage via labile phases, such as working and short-term memory to the stable form of long-term memory. Thus it was shown, that** s**everal drugs inhibited short-term memory without altering long-term memory [34] and that working, short-term, and long-term memory were differentially regulated in the various brain regions by the various neurotransmitter systems, including cholinergic one [34–37]. The authors concluded that different types of memory had the separate mechanisms, various neurotransmitter systems, and regions. These data demanded an individual approach for the restoration of cognitive abilities.

The basis of our investigation is importance of the cholinergic systems in human and animal cognition, and also the existence of general mechanisms in the development of dementias of different aetiologies. In the present study, the cholinergic synaptic organization of different forms of learning and memory in rats with normal and chronic ischaemic brains was investigated using spatial contextual and spatial cued models in the Morris water maze. A marker of cholinergic neurons, enzyme of acetylcholine synthesis choline acetyltransferase (ChAT; EC 2.3.1.6) was used for estimation of the cholinergic systems. ChAT activity and also protein contents (total synaptic parameters) were measured in subfractions of the synaptic membranes and the synaptoplasm isolated from “light” and “heavy” synaptosomal fractions of the cortex and the hippocampus. Thus, the participation of projective and intrinsic cholinergic systems of the cortex and hippocampus in mechanisms of learning and memory under normal and ischaemic conditions was researched. In addition, the regulation of learning performance under prolonged action of selective agonist of *α*4*β*2 subtype of nAChR metanicotine (RJR) and selective antagonist of non*α*7 subtypes of nAChR mecamlamine was studied.

## 2. Materials and Methods

### 2.1. Animals

Outbred white adult male rats (220–270 g) were supplied from the animal's nursery “Light mountains” (Russia) and then kept in the vivarium of our Institute of General Pathology and Pathophysiology. The rats were housed in a temperature-controlled room (20–24°C) with free access to food and water and kept on a 12 h light/dark cycle according to the National Institutes of Health Animal Care and the “Principles of Laboratory Animal Care” guidelines and the study was approved by the Ethical Committees of the Institutes. All animals were allocated to experimental groups randomly, using random numbers.

### 2.2. Chronic Ischaemia Model (Two-Vessel Occlusion)

Chronic rat ischaemia was induced by permanent occlusion of the common carotid arteries (two-vessel occlusion, 2VO) by ligation. The bilateral common carotid arteries were tied with silk threads whilst the rats were under an appropriate level of pentobarbital anaesthesia. The common carotid arteries were separated from the cervical sympathetic and vagal nerves through a ventral cervical incision. The sham-operated animals (control groups) underwent a similar surgery but vessel ligation was excluded.

### 2.3. Behaviour Tests

Behaviour was studied in spatial contextual (noncued) or spatial cued models of learning and memory in the Morris water maze following standard procedures [38]. The experimental apparatus consisted of a circular water pool (diameter, 120 cm; height, 60 cm) filled with milk-clouded water at 24°C to a depth of 40 cm. A Plexiglas hidden platform (10 × 10 cm) was submerged 2 cm below the water surface and was placed at the midpoint of one quadrant. 

Rat training started 6, 7, or 8 days after the surgery. The rats were trained during three daily sessions in the contextual (sham-operated/2VO rats) or the cued learning models. In both learning models the rats were given four daily attempts to find the hidden platform in a 60 s time interval and the estimated swim time for platform achievement (latency time) was recorded. Rats which failed to find the platform within 60 s were considered unable to solve the task and were softly guided there by the investigator with 60 s scored. The other rats remained on the platform for 30 s and were returned to their home cage during the intertrial interval (60 s).

In the contextual model the location of the hidden platform remained the same throughout the training period. The pool was located in a test room containing no prominent visual marks. At the start of all trials, the rats were placed in the pool at one of four starting positions. In the cued model a prominent visual mark (cue) was placed on the maze wall over the hidden platform to help the animal locate the platform. In this model the rats had the same starting position but the hidden platform with its cue was moved to four different positions during the session.

 The following forms of cognitive functions were observed and investigated: the inherited abilities (the first noncasual attempts at decision making in the task, that is, 1s1 trial in the cued model and 1s2 trial in the contextual model); working memory in the first session (1s2–4 and 1s3-4 averaged out over the following trials, resp.); learning in the second and the third sessions (2s2–4 and 3s2–4 averaged trials, resp.); and long-term memory on the days after the first and the second sessions of training (2s1 and 3s1 trials, resp.). 

 All behavioural experiments were carried out by investigators who had no knowledge of the experimental groups.

### 2.4. Drug Administration

Metanicotine (RJR 2304, Tocris), a selective agonist of *α*4*β*2 subtype of nAChR and mecamilamine (Sigma), a selective antagonist of non-*α*7 subtypes of nAChR, were used. The preparations were subchronically administered (i.p.) three times daily in doses of 26 and 3.9 nmoles/kg, respectively. Both the sham-operated and the ischaemic rats received the first injection of the preparations immediately after the end of narcosis (1.5–3 hours after surgical intervention). The control group of the sham-operated animals received saline. The rats were tested 6–10 days after 2VO.

### 2.5. Brain Tissue Preparation

Some of animals were decapitated for biochemical analysis 3-4 days after the third session of training. It means, the rats which trained from 6 day after the surgery were decapitated at 11 or 12 days after the surgery and so on. The biochemical group included the control/2VO animals trained in the contextual model (contextual biochemical subgroup) or the cued model (cued biochemical subgroup).

All preparative procedures were carried out at 2–4°C. Briefly, the brain, cortex and hippocampus were removed, separated and homogenized. From each sample the light and heavy synaptosomal fractions were isolated, with further separation of the subfractions of the synaptic membranes and the synaptoplasm, following preparative and the disruptive procedures and the discontinued gradients of sucrose density as described previously [30, 39]. The fractions of the synaptosomes were obtained from the rough mitochondrial fraction by centrifugation using a bucket rotor (84,000 g × 120 min, 2–4°C) in layers between 1.0–1.2 M sucrose densities (the light synaptosomes) and between 1.2–1.4 M sucrose densities (the heavy synaptosomes) [40]. The synaptosomes were disrupted by combined shock procedures: the synaptosome pellets were suspended in hypo-osmotic solution containing 6 mM Tris-NCl buffer, pH 8.1 [41] (100 mg tissue/ml) and they were then exposed by freeze-thawing. The synaptoplasm subfractions were obtained as supernatants by centrifugation from the disrupted synaptosomal fractions (14,000 g × 30 min, 2–4°C). The pellets were suspended in the hypo-osmotic solution and stratified on discontinued gradients again. The synaptic membrane subfractions were obtained by centrifugation using the bucket rotor (130,000 g × 120 min, 2–4°C) in layers between 0.6–1.2 M sucrose densities. The clean synaptic membrane subfractions were free from glial, mitochondrial and synaptic vesicle contamination [39]. All samples were stored at −70°C until the day of the assay.

### 2.6. Analytical Methods

The activity of ChAT in subfractions of synaptic membranes and synaptoplasm of the cortex and the hippocampus was determined by the radiometric method of Fonnum [42] and the protein contents were determined by the method of Lowry et al. [43]. Accordingly, the membrane-bound mChAT activity and m-protein contents were determined in the synaptic membrane subfractions, and the water-soluble cChAT activity and c-protein contents were estimated in synaptoplasm subfractions. Moreover, both the light and the heavy synaptosomal fractions include presynapses of different sizes [38]. Small and large presynapses have different relationships with synaptic membranes and synaptoplasm mass. This is the reason why changes in mChAT activity could be exposed and changes in cChAT activity could be masked in the small presynapses, whereas changes in cChAT activity, but not in mChAT activity, could be exposed in the large presynapses. Therefore, estimations of mChAT and cChAT activities (as well as m- and c-protein contents) could give additional information on the characteristics of changes caused by ischaemia.

#### 2.6.1. Choline Acetyltransferase Assay

The reactive solution was prepared at the day of experiment. The enzymatic reaction was started by mixing subfraction samples with the reactive solution. The reactive mixture contained a final concentration of 0.2 mM acetyl CoASA (Fluka) and [1-^14^C]-acetyl CoASA (Amersham Pharmacia Bioscience) with SPA 5 mCi/mmol, 300 mM NaCl, 3 mM MgCl_2_, 0.2 mM physostigmine salicylate (Sigma), 10 mM choline chloride (Serva), 0.5% Triton X-100 (Serva), 0.5 mg/ml albumin from bull serum (Koch-Light), 10 mM sodium phosphate buffer/1 mM EDTA-Na_2_, pH 7.8 and the subfraction samples (near 3.5 mg of protein) at a common volume of 0.05–0.1 ml. The reactive mixture was incubated in a water shaker at 37°C for 30–60 min. The reaction was stopped by adding 2 ml of ice-cold stop solution (0.2 mM acetylcholine in 10 mM sodium phosphate buffer/1 mM EDTA-Na_2_, pH 7.8) and by placing the mixture in an ice bath. Then, a 1 ml solution of sodium tetraphenylborate (Sigma) in butyl acetate (15 mg/ml) was added and quickly subjected to intensive mixing in a shaker (500 turns/min, 4 min, room temperature). The organic phase was separated from the inorganic phase by centrifugation (1000 g × 15 min, 2–4°C). The organic phase with acetylcholine (0.5–0.7 ml) was placed into scintillation liquid for organic solutions and the radioactively synthesized acetylcholine (DPM) was quantified with a Beta counter.

#### 2.6.2. Protein Assay

Reactive solution (Biuret reagent) was prepared at the day of experiment by mixing 0.5 ml of 1% cupric sulfate with 0.5 ml of 2% sodium potassium tartrate, followed by the addition of 50 ml of 2% sodium carbonate in 0.1 N NaOH. A standard curve was prepared as follows. Bovine serum albumin (BSA) powder was dissolved in distilled water and diluted to a concentration of 1000 *μ*g/ml. A series of dilutions of the basic BSA solution (50, 100, 200, 400 and 500 *μ*g/ml) was made by mixed thoroughly of the aliquots of basic BSA solution and water with repeated pipeting. Samples were within the BSA standard range (1–20 *μ*g in assay volume). Reaction was started by intensive mixed of 0.02/0.04 ml of BSA or subfractions samples with 1 ml of the reactive solution. The mixture was then allowed to incubate at room temperature for 10–15 min prior to the addition of 0.1 ml per tube of 1.0 N Folin & Ciocalteu's reagent. Samples were mixed immediately. Color was allowed to develop for 2 hours in dark at room temperature and the absorbance of the reduced Folin reagent measured at 750 nm and blanked on the water only control. After then the reaction was found to be stable for up to an hour at room temperature and kept in refrigerator at 5–8°C for up to 1-2 days.

### 2.7. Statistical Analysis

The behavioural results were expressed in terms of time taken to swim to the hidden platform (s) and the biochemical results were expressed in terms of ChAT activity (nmoles acetylcholine/min) or protein content (mg) in 1 g wet weight of cortex and hippocampus tissue, respectively. The data were calculated using the nonparametric Fisher's Exact Test and the *r*-criterion of the Pearson's correlative test in Microsoft Excel with a glance of adjusting formula for small number of observations [44]. Differences were considered to be statistically significant if *P* < .05.

## 3. Results

### 3.1. Behavioural Performance under 2VO Conditions: Total the Contextual and the Cued Groups

**Figure 1:**
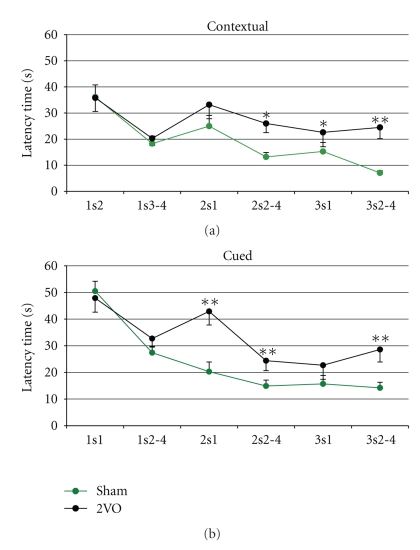
Behavioural performance of control and ischaemic rats in the Morris water maze. Graphs represent the swim latency time (sec, mean ± SEM) of the hidden platform achievement (behavioural criterion) by control (sham-operated, green curve) and ischaemic (2VO-operated, black curve) rats in spatial contextual (on the top) and spatial cured (on the bottom) behavioural conditions.

**Figure 2:**
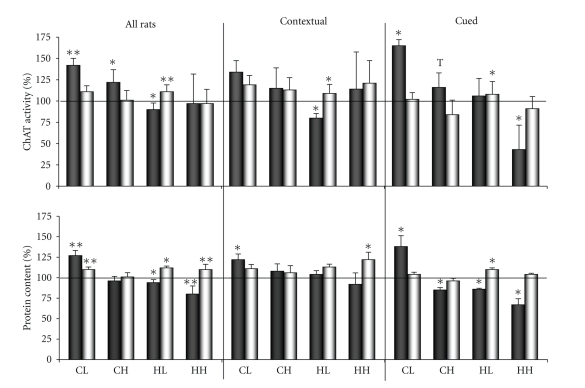
ChAT activity (top row of bars) and protein content (bottom row of bars) in the subfractions of the synaptic membranes and synaptoplasm of the light and heavy synaptosomes of the cortex and the hippocampus under 2VO conditions. Over the bars: All Rats, whole rat biochemical groups (*n* = 9 for control and 2VO rats); Contextual: the spatial contextual biochemical rat's subgroup (*n* = 5 per group); Cued: the spatial cued biochemical rat's subgroup (*n* = 4 per group). All values were expressed as percentage of enzyme activity or protein content in comparison to their control values (100%). For each pair of bars: dark bar represents synaptic membrane subfraction; light bar: synaptoplasm subfraction. CL: the subfractions of the light synaptosomal cortical fraction; CH: the subfractions of the heavy synaptosomal cortical fraction; HL: the subfractions of the light synaptosomal hippocampal fraction; HH: the subfractions of the heavy synaptosomal hippocampal fraction. *,**, significant differences from control (*P* < .05 and *P* < .025, resp.) by the Fisher's Exact test. Comments and *r*-criterion values of the significant correlations by the Pearson's test between ChAT activity and protein content or of the significant correlations between m- and c-parameters in own synaptosomal fractions see in Section 3.

The period of 6–10 days of chronic brain ischaemia led to a strong decline in training efficiency in the Morris water maze. Learning and long-term memory were impaired in both the contextual and cued models (Figure 1). Learning in 2s2–4 and 3s2–4 were impaired in a similar manner in both of the behavioural models, whereas impairment of the long-term memory had the specificity in each model. In the contextual model (Figure 1, top), impairment of long-term memory developed gradually and only 3s1 was significantly impaired. In the cued model (Figure 2, bottom), long-memory 2s1 was impaired and 3s1 was the same as the control. Working memory and inherited abilities were intact in both behavioural models. 

It can be noted that although the investigated cognitive functions were impaired, they were still performed in ischaemic rats. From all of the investigated animals (*n* = 27), only two rats could not solve the tasks in our experimental conditions (in the contextual model). As a rule, the prolongation of solving tasks and/or the delay in learning (successful solving of the task only occurred in the third session) was observed.

### 3.2. ChAT Activity and Protein Content under 2VO Conditions. Total Biochemical Group

The period of 11–14 days of chronic ischaemia resulted in significant changes in ChAT activity and protein content in the investigated subfractions of the synaptosomes, both of the cortex and the hippocampus (Figure 2-all rats). In the cortical light synaptosomal fraction, mChAT activity and m-protein content were increased, and these changes were positively correlated amongst themselves (*r* = +0.770, *n* = 18, total control and 2VO groups data, *P* < .001; in the control group *r*   = + 0.788, *n* = 9, *P* < .02). The cChAT activity did not significantly vary or correlate with mChAT activity but it was positively correlated with increasing c-protein content (*r* = +0.669, *n* = 9, *P* < .05; in the control group *r* = +0.305, *n* = 9, *P* > .05). This indicated a reorganization of the synaptic pool in more than one synaptic population of the cholinergic projective neurons in the cortex. 

The activity of mChAT activity was only increased in the cortical heavy synaptosomal fraction. But mChAT activation was accompanied by a reinforcement in the positive correlation between its values and the m-protein content (*r*   = + 0.694, *n* = 9, *P* < .05; in the control group *r* = +0.291, *n* = 9, *P* > .05). Also, a reinforcement of the positive correlation between the values of cChAT activity and c-protein content was observed (*r* = +0.835, *n* = 9, *P* < .01; in the control group *r* = +0.633, *n* = 9, *P* > .05). At the same time, the correlation between the activity of mChAT and the activity of cChAT became weaker than in the control (*r* = +0.579, *n* = 9, *P* > .05; in the control group *r* = +0.754, *n* = 9, *P* < .02). This indicated a reorganization of the synaptic pool in more than one synaptic population of the cholinergic interneurons in the cortex. 

In the hippocampal light synaptosomal fraction, mChAT activity decreased and cChAT activity increased. The protein content showed similar changes. However, significant correlations between the values of mChAT and cChAT activity, and between the values of ChAT activity and protein content, were absent. This indicated a reorganization of the synaptic pool in more than one synaptic population in the hippocampus, in both the cholinergic systems and some noncholinergic systems. 

In the hippocampal heavy synaptosomal fraction, m-protein content decreased and c-protein content increased. Changes in the m-protein content did not correlate with ChAT activity and thus reflected reorganization of noncholinergic presynapses in the hippocampus. The activities of mChAT and cChAT did not differ from the controls although at the same time a positive correlation arose between their values (*r* = +0.802, *n* = 8, *P* < .02; in the control group *r* = −0.300, *n* = 9, *P* > .05). Also, the positive correlations between values of c-protein content and mChAT activity (*r* = +0.927, *n* = 8, *P* < .01; in the control group *r* = +0.265, *n* = 9, *P* > .05) and cChAT activity (*r* = +0.844, *n* = 8, *P* < .01; in the control group *r* = +0.091, *n* = 9, *P* > .05) were reinforced. This indicated a reorganization of the presynapses of the cholinergic interneurons/lateral pathway projective neurons in the hippocampus. It seems that the unchanged values of ChAT activity reflected parallel processes of activation and inactivation of ChAT in different synaptic populations of this fraction.

### 3.3. ChAT Activity and Protein Content in the Contextual and Cued Biochemical Subgroups

Also, the biochemical data of the contextual biochemical subgroup were compared with the cued biochemical subgroup. Analysis of the biochemical parameters in the control biochemical subgroups did not reveal significant changes between the subgroups (Table 1). Only the values of mChAT activity were lower in the cortical heavy and in the hippocampal light synaptosomal subfractions in the cued biochemical subgroup of rats as compared with the contextual one. 

Analysis of the biochemical parameters in the 2VO biochemical subgroups confirmed our observations about the reorganization of the synaptic pool. In the cortical light synaptosomal fraction in the contextual biochemical subgroup (Figure 2-contextual), independent correlations were detected between the values of mChAT activity and m-protein content (*r* = +0.765, *n* = 10, total control and 2VO contextual biochemical subgroups data, *P* < .01; in the control contextual biochemical subgroup *r* = +0.774, *n* = 5, *P* > .05) and between the values of cChAT activity and c-protein content (*r* = +0.987, *n* = 5, *P* < .01; in the control contextual biochemical subgroup *r* = +0.441, *n* = 5, *P* > .05). In the cued subgroup (Figure 2-cued), a correlation was only detected between mChAT activity and m-protein content (*r* = +0.783, *n* = 8, total control and 2VO cued biochemical subgroups data, *P* < .05; in the control cued biochemical subgroup *r* = +0.622, *n* = 4, *P* > .05) and a correlation was revealed between mChAT and cChAT activities (*r* = +0.995, *n* = 4, *P* < .01; in the control biochemical cued biochemical subgroups *r* = −0.404, *n* = 4, *P* > .05).

In the cortical heavy synaptosomal fraction in the contextual biochemical subgroup, independent correlations were detected between mChAT activity and m-protein content (*r* = +0.981, *n* = 5, *P* < .01) and between the values of cChAT activity and c-protein content (*r* = +0.966, *n* = 5, *P* < .01), whereas there was no correlation between the activities of mChAT and cChAT among themselves (*r* = +0.652, *n* = 5, *P* > .05). In the cued biochemical subgroup, an increase in mChAT activity was detected (a tendency) whereas a decrease in m-protein content was revealed. The decrease in m-protein content allows to suppose the changes in noncholinergic presynapses. 

In the hippocampal light synaptosomal fraction in the contextual biochemical subgroup, an independent decrease in mChAT activity and an increase in cChAT activity only, and in the cued biochemical subgroup an increase in cChAT activity only, and a decrease in m-protein content and an increase in c-protein content were detected. 

In the hippocampal heavy synaptosomal fraction in the contextual biochemical subgroup, positive correlations were detected between the increased values of c-protein content and mChAT activity (*r* = +0.922, *n* = 5, *P* < .01) and cChAT activitiy (*r* = +0.919, *n* = 5, *P* < .05), and between mChAT and cChAT activities (*r* = +0.910, *n* = 5, *P* < .05; in the control contextual biochemical subgroup *r* = −0.266, *n* = 5, *P* > .05). However, in the cued biochemical subgroup, a decrease in mChAT activity and m-protein content was revealed and these changes did not correlate among themselves. This indicated a reorganization of the presynapses of the cholinergic interneurons/lateral pathway projective neurons and noncholinergic neurons in the hippocampus.

### 3.4. Comparison of the Behavioural Performance and ChAT Activity in the Control Rats of the Biochemical Subgroups

**Figure 3:**
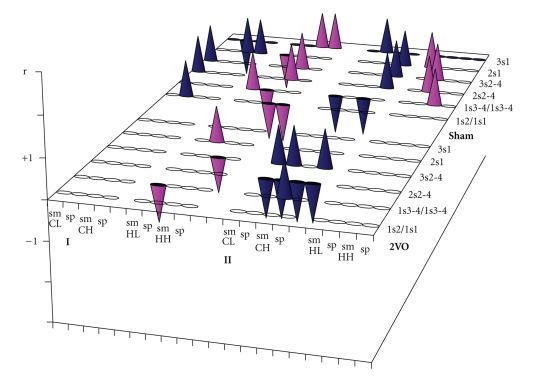
Significant *r*-criterion values (by the Pearson's test) of behavioural performance and ChAT activity in rats in the Morris water maze in the spatial contextual (I) and the spatial cued (II) behavioural models under control (sham) and ischaemic (2VO) conditions. 1s2/1s1, 1s3-4/1s2–4, 2s2–4, 3s2–4, 2s1, 3s1 are the same as in Figure 1; sm and Sp, subfractions of the synaptic membranes and the synaptoplasm, respectively, of the cortical light (CL) and heavy synaptosomes (CH) and hippocampal light (HL) and heavy synaptosomes (HH) as in Table 1. In the spatial contextual biochemical subgroup *n* = 5 for control and 2VO rats; in spatial cued biochemical subgroup *n* = 4 for control and 2VO rats.

Differentiation of the rats into biochemical subgroups, tested in the contextual and cued models, permit to compare the behavioural performance and ChAT activity in these rats. Under the normal conditions, each form had individual cholinergic composition (Table 2: sham-contextual, Figure 3-I: sham). The inherited ability 1s2 cholinergic composition included large presynapses of projective cortical neurons (positive correlation with cChAT activity) and presynapses of hippocampal interneurons/lateral pathway projective neurons (negative correlation with mChAT and cChAT activities). The same cholinergic structures associated with the long-term memory 3s1, but with inverse symbols of *r*-criterions. The long-term memory 2s1 had composition other than 3s1 which involved small presynapses of the projective hippocampal neurons (negative correlation with mChAT activity) and some populations of hippocampal interneurons (positive correlations with mChAT and cChAT activities). The composition of the working memory 1s3-4 involved small presynapses (positive correlation with mChAT activity) and large presynapses (negative correlation with cChAT activity) of the projective hippocampal neurons. The composition of learning 2s2–4 and 3s2–4 was identical and involved small presynapses of the projective cortical neurons (positive correlation with mChAT activity in both forms of learning) and the hippocampal interneurons/lateral pathway projective neurons (positive correlations with mChAT activity). 

 The similar analysis in the cued biochemical subgroup revealed other individual cholinergic compositions of learning and memory (Table 2: sham-cued, Figure 3-II: sham). According to our data, the cholinergic systems did not participate in realization of inherited abilities 1s1. Working memory 1s2–4 composition involved small presynapses of both cortical cholinergic systems (negative correlation with mChAT activity) and of the hippocampal interneurons/lateral pathway projective neurons (positive correlation with mChAT activity). The composition of learning 2s2–4 and 3s2–4 was identical and comprised large presynapses of cortical interneurons and projective hippocampal neurons (positive correlations with cChAT activity in both cases). The long-term memory composition in 2s1 involved large presynapses of the cortical interneurons and small presynapses of projective hippocampal neurons (in both cases, there were positive correlations with cChAT or mChAT activities). And the long-term memory composition in 3s1 involved large presynapses of the cortical projective neurons (positive correlations with cChAT activity).

### 3.5. Comparison of the Behavioural Performance and ChAT Activity in the Ischemic Rats of the Biochemical Subgroups

Chronic brain ischaemia had considerable effects on the cholinergic organization of the investigated cognitive functions. In the contextual model (Table 2: 2VO-contextual, Figure 3-I: 2VO), inherited abilities 1s2, learning 2s2–4 and long-term memory 3s1 completely lost correlations with the cholinergic populations and all forms of cognition lost correlations with the cortical cholinergic populations. The composition of working memory 1s3-4 only kept negative connections with the large presynapses of projective hippocampal neurons. The positive connections of learning 3s2–4 with the small presynapses of the hippocampal interneurons/lateral pathway projective neurons inversed to negative ones. The long-term memory composition 2s1 consisted of only new, positive connections with large presynapses of the projective hippocampal neurons. 

 In the cued model (Table 2: 2VO-cued, Figure 3-II: 2VO), long-term memory 2s1 and 3s1 completely lost correlations with the cholinergic populations and all forms of cognition lost correlations with the hippocampal cholinergic influences. The working memory 1s2–4 lost its negative connections with the small presynapses of cortical projective neurons and its negative connections with the small presynapses of cortical interneurons inversed to positive ones. The composition of learning 2s2–4 included a reversal to negative connections with the large presynapses of cortical interneurons, new negative connections with presynapses of cortical projective neurons and new negative connections with the small presynapses of cortical interneurons. The learning composition 3s2–4 kept its connections with the large presynapses of cortical interneurons and added new negative connections with presynapses of the cortical projective neurons.

So, under 2VO conditions as in the contextual and in the cued biochemical subgroup quantity of the cholinergic connections with the cognitive functions significantly reduced and some new links arose. Each form of cognition as resulting had 2VO cholinergic synaptic composition organized differently from normal ones.

### 3.6. Analysis of Correspondence between 2VO Induced Changes in Behavioral Performance and ChAT Activity in the Key Cholinergic Populations in the Biochemical Subgroups

**Figure 4:**
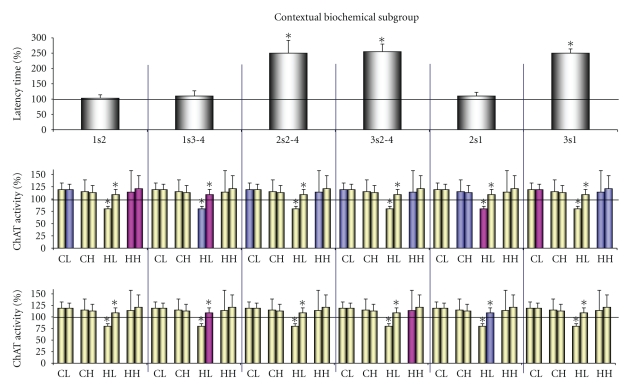
2VO induced changes in behavioral performance and ChAT activity in the key cholinergic populations in the spatial contextual biochemical subgroup (*n* = 5). 1s2, 1s3-4, 2s2–4, 3s2–4, 2s1 and 3s1, the same forms of cognition as in Figure 1. Behavioral performance was expressed as percentage of latency time compared to their control values (100%). CL, CH, HL, HH, the same pairs of the subfractions as in Figure 2. Values of ChAT activity in the subfractions are identical to ones in Figure 2-contextual, and they were duplicated under the each form of cognition. Blue bars, the values of ChAT activity had positive significant correlations with corresponding forms of cognition; violet bars, the values of ChAT activity had negative significant correlations with corresponding forms of cognition. In the middle row were marked correlations between behavioral performance and ChAT activity in the control rats (correlations correspond to Table 2, Sham-Contextual). In the bottom row were marked correlations between behavioral performance and ChAT activity in 2VO rats (correlations correspond to Table 2, 2VO—Contextual). *, *P* < .05 by the Fisher's exact test. See comments in Section 3.6.

We attempted to analyze the dependence of impairment of the investigated cognitive functions in 2VO conditions from the reorganization of key cholinergic systems. It seems, in the contextual biochemical subgroup only preservation of the inherited abilities 1s2 from damage could be explaned by preservation of the key cholinergic populations, revealed in the normal conditions (Figure 4, middle row). But long-term memory 3s1 had the same cholinergic composition with inverse symbols of *r*-criterions. In this case it would be expected that 3s1 would also be protected; however, this did not take place. Moreover, according to the cholinergic organization under normal conditions, working memory 1s3-4 would be considerably impaired, whereas long-term memory 2s1 and learning 2s2–4 and 3s2–4 would be considerably improved; however, these did not occur either. 

On the other hand, the new cholinergic composition in 2VO conditions had accordance between reinforcement of the negative influence of the hippocampal interneurons/lateral pathway projective neurons on learning 3s2–4 and impairment of this function (Figure 4, bottom row). But reinforcement of new negative and positive influences of projective hippocampal neurons was not reflected in the performance of either working memory 1s3-4 or long-term memory 2s1. 

**Figure 5:**
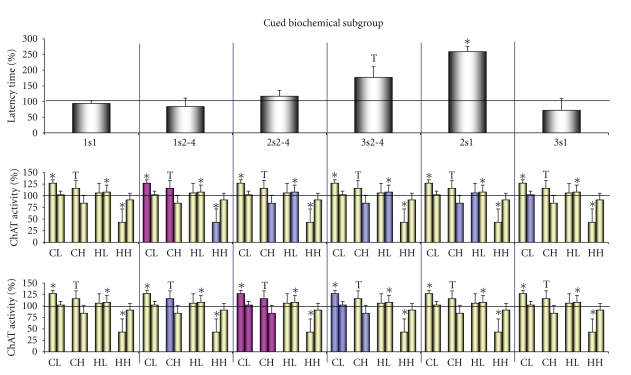
2VO induced changes in behavioral performance and ChAT activity in the key cholinergic populations in the spatial cued biochemical subgroup (*n* = 4). 1s2, 1s3-4, 2s2–4, 3s2–4, 2s1 and 3s1, the same forms of cognition as in Figure 1. Behavioral performance was expressed as percentage of latency time compared to their control values (100%) as in Figure 4. CL, CH, HL, HH, the same pairs of the subfractions as in Figure 2. Values of ChAT activity in the subfractions are identical to ones in Figure 2-cued, and were duplicated under the each form of cognition as in Figure 4. Blue and violet bars the same as in Figure 4. In the middle row were marked correlations between behavioral performance and ChAT activity in the control rats (correlations correspond to Table 2, Sham-Cued). In the bottom row were marked correlations between behavioral performance and ChAT activity in 2VO rats (correlations correspond to Table 2, 2VO—Cued). *, *P* < .05 by the Fisher's Exact test. See comments in Section 3.6.

In the cued biochemical subgroup, only the preservation of long-term memory 3s1 could be explained by the resistance to ischaemia of the key synaptic population revealed in normal conditions (Figure 5, middle row). At the same time, working memory 1s2–4 would be considerably impaired, learning 2s2–4 and 3s2–4 would be equally improved or otherwise unchanged and long-term memory 2s1 would be unchanged, but these were not observed.

 On the other hand, the absence of 1s2–4 impairment could also be explained by the new, weakly expressed positive cholinergic influence (tendency) of the large presynapses of cortical interneurons (Figure 4, bottom row). Then the distinctions in learning 2s2–4 and 3s2–4 performance in 2VO conditions would be explained by their new cholinergic compositions if we suppose more considerable influence of the large presynapses of the cortical interneurons on these functions in comparison with the influence of the other new key synaptic populations. Such supposition is in accordance with the data regarding long-term memory 2s1. The impairment of 2s1 also would be explained by the reduction of the link with this key synaptic population under the normal conditions. 

So, it seems that performance of the cognitive functions as in the contextual and in the cued model under 2VO conditions, as a rule, did not depend on their cholinergic organization, revealed in normal conditions. Contrary, new cholinergic organization, revealed in 2VO conditions showed more significant correlations with changes in behavioral performance.

### 3.7. Regulation of the Learning Performance in the Normal and 2VO Conditions by Selective nAChR Agonist RJR and Antagonist Mecamilamine

Whereas, the contextual and cued learning 2s2–4 and 3s2–4 revealed cholinergic synaptic compositions identical for normal conditions and different ones, revealed in 2VO conditions, it was investigated prolonged action on the learning performance of the selective agonist of *α*4*β*2 subtype of nAChR RJR and the selective antagonist of non-*α*7 subtypes of nAChR mecamilamine. 

**Figure 6:**
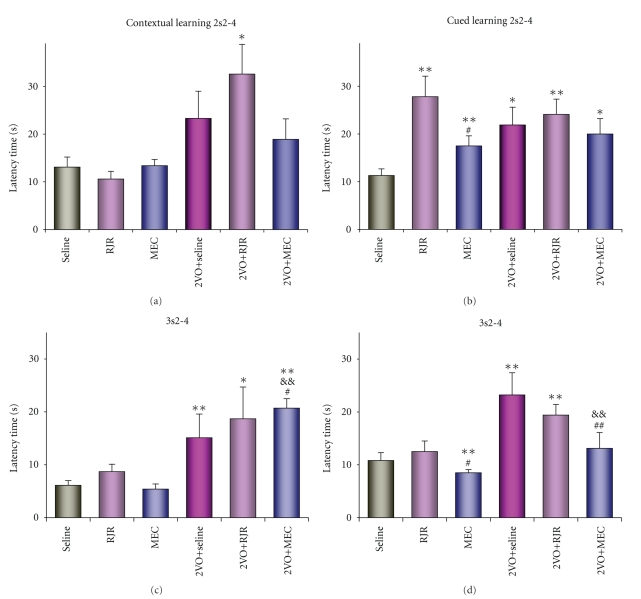
Influence of the agonist of *α*4*β*2 subtype of nAChR RJR and the antagonist of non-*α*7 subtype of nAChR mecamilamine (MEC) on learning 2s2–4 (top row of figures) and 3s2–4 (bottom row of figures) in rats in the Morris water maze in the spatial contextual and the spatial cued models under control and 2VO conditions. The learning performance is presented as latency time (sec). Seline, control rat's groups (*n* = 12 for learning as 2s2–4 and 3s2–4 in the contextual model; *n* = 13 for the ones in the cued model); RJR, MEC, groups of rats with RJR or MEC administered under the normal conditions (*n* = 6 for both groups for learning as 2s2–4 and 3s2–4 in the contextual model; *n* = 7, 11 for learning 2s2–4 in RJR, MEC groups, respectively, and *n* = 10, 11 for learning 3s2–4 in ones in the cued model); 2VO + seline, the 2VO rat's group (*n* = 9 for learning as 2s2–4 and 3s2–4 in the contextual model; *n* = 7 for the ones in the cued model); 2VO + RJR, 2VO + MEC, groups of rats with RJR or MEC administered under the 2VO conditions (resp., *n* = 9, 10 for learning as 2s2–4 and 3s2–4 in the contextual model; *n* = 8, 9 for the ones in the cued model). *, **, significant differences from control rat's group (*P* < .05 and *P* < .025, resp.); ^#^,^# #^, significant differences between RJR and MEC or 2VO + RJR and 2VO + MEC rat's groups (*P* < .05 and *P* < .025, resp.); &&, significant differences between 2VO and 2VO + MEC rat's groups (*P* < .025) by the Fisher's Exact test.

In the contextual model under normal conditions, both the agonist RJR and the antagonist mecamilamine did not influence on learning as 2s2–4 and 3s2–4 performance. It seems, this fact indicate that non-*α*7 subtypes of nAChR did not participate in the regulation of ones (Figure 6, contextual learning). under the 2VO conditions, effects of RJR on both learning performance was absent similar whereas mecamilamine potentiated effect of 2VO on learning 3s2–4. Evidently, the *α*4*β*2 subtype did not participate in the regulation of the contextual learning, as before, while some non-*α*7 and non-*α*4*β*2 subtypes participated with negative influences on learning 3s2–4.

In the cued model under normal conditions, the agonist RJR impaired learning 2s2–4 and did not affect learning 3s2–4 (Figure 6, cued learning). The antagonist mecamilamine impaired learning 2s2–4 and improved learning 3c2–4. The negative effect of the agonist on learning in 2s2–4 was significantly greater than that of the antagonist (*P* < .05). The difference between the agonistic and antagonistic actions on learning in 3s2–4 at were also significant (*P* < .05). It follows that the *α*4*β*2 subtype (negative influence) and some non-*α*7 and non-*α*4*β*2 subtypes of nAChR (positive but weak influence) participated in the regulation of learning in 2s2–4. At the same time, the *α*4*β*2 subtype did not participate in the regulation of learning 3s2–4, while some non-*α*7 and non-*α*4*β*2 subtypes participated with negative influences. under the 2VO conditions, RJR did not correct the impaired functions 2s2–4 and 3s2–4, and mecamilamine did not correct learning 2s2–4 but resulted in normal learning 3s2–4 performance. It seems, non-*α*7 subtypes of were removed from the receptor composition of learning 2s2–4. At the same time, the importance of some non-*α*7 and non-*α*4*β*2 subtypes of nAChR was reinforced or new connections arose in the receptor organization of learning 3s2–4 (negative influence). 

So, in the contextual learning 2s2–4 and 3s2–4, nAChR were absent in normal and were acquired in 2VO receptor composition (3s2–4). Then, the cued learning 2s2–4 and 3s2–4, with identical cholinergic synaptic compositions in the norm, had differences in receptor compositions. Moreover, the cued learning 2s2–4 and 3s2–4 had also differences in 2VO receptor compositions via another means.

## 4. Discussion

### 4.1. Characteristic of the Chronic 2VO Brain Ischaemia Influence on the Rat's Behavioural Performance in the Morris Water Maze and the Cortical and Hippocampal Cholinergic Synaptic Pool

This research showed that the chronic 2VO brain ischaemia model was an efficient model of neurodegenerative disorders, which was the first purpose of our investigation. The period of 6–10 days of 2VO ischaemia provoked typical attributes of vascular dementias such as impairment of learning and long-term memory in both spatial-contextual and spatial-cued models of behaviour in the Morris water maze. The inherited abilities and working memory remained intact, and damage to the cued long-term memory was transient in this ischaemic period. A considerable reorganization of the synaptic pool of all investigated cholinergic systems in the cortex and the hippocampus was revealed in these same 2VO rats 11–14 days after the surgery. A decrease in mChAT or cChAT activity in one synaptosomal fraction and an increase in another were obtained as result of the 2VO influence, but not of the training, whereas the biochemical parameters did not reveal similar changes between the control contextual and cued biochemical subgroups except one. It is possible that decrease in mChAT activity in the hippocampal heavy synaptosomal fraction in the cued biochemical subgroup was result of the training (see Table 1 and Figure 2-cued).

The decrease in mChAT and cChAT activity reflected cholinergic hypofunction or a degeneration of the cholinergic presynapses. Neurodegeneration was observed in different brain ischaemia models starting from the second day up to half a year of ischaemia [45, 46]. Dysfunction of ChAT in the projective fibres in the hippocampus (representing 80–90% of the total activity of this enzyme, [30] and see Table 1) was described at 7–14 days of ischaemia [6, 13, 14, 47, 48]. It was shown in vitro that the activity of mChAT was selectively suppressed when the exchange of acetylcholine was damaged by inhibition of the vesicular acetylcholine transporter [49] or the high affinity transport of choline [50]. The function of the vesicular acetylcholine transporter depends on the proton gradient, which in turn is disturbed due to falling ATP levels (inhibition of the proton ATPase) or acidosis [49]. The chronic ischaemia/hypoxia provokes both these factors [51–53]. One effect of the degenerative process is a decrease in protein content. We did not reveal a decrease in protein content correlated with ChAT activity in our research. But we did suppose that the correlation between ChAT activity and protein content could be masked because of the complex opposing processes that took place in some of the synaptosomal fractions. 

At the same time, according to data in the literature, sprouting and destruction with the swelling of neurons and their terminals predominates in late brain ischaemia or postischaemic reoxygenation (in days and months) [3, 48, 53, 54]. In our research, activation of ChAT was also observed in the majority of the synaptic subfractions, and it could have reflected cholinergic hyperfunction or synaptogenesis (sprouting). It is known that synaptic hyperfunction is accompanied with an enhanced structuring of proteins from the synaptoplasm. Under these conditions, the m-protein content will increase and the c-protein content will decrease. Therefore, the correlated increase between mChAT activity and m-protein content will be reflected as cholinergic hyperfunction and synaptogenesis (cortical light synaptosomes in the biochemical total group and both subgroups), whereas the correlated increase between cChAT/mChAT activity and the c-protein contect will only reflect synaptogenesis (cortical light synaptosomes in the biochemical total group and the contextual subgroup, hippocampal heavy synaptosomes in the total group and the cued subgroup). Selective activation of mChAT in vitro was shown under conditions of impaired ionic balance such as an accumulation of [Ca^2+^]_*i*_ and [Zn^2+^]_*i*_ [55, 56]. It was revealed that [Zn^2+^]_*i*_ precedes [Ca^2+^]_*i*_ accumulation [56] and [Ca^2+^]_*i*_ in turn results in the functional hyperactivation and swelling of synapses [53, 57, 58]. According to data in vitro activation of cChAT reflect cholinergic hyperfunction under normal ionic and metabolic conditions [49, 59, 60]. cChAT activation under ischemic pathology did not describe in the literature. Therefore, we suppose that the activation of cChAT reflected synaptogenesis, in agreement with other researchers [3]. 

Thus, chronic brain ischaemia for 11–14 days resulted in a complex reorganization of the cortical and hippocampal synaptic pool which involved synaptogenesis or hyperfunctions in the unbalanced ionic conditions of one of the cholinergic synaptic populations and degeneration or dysfunction of the others. We supposed that all of the cholinergic processes revealed under 2VO conditions were present in both cholinergic subgroups of the rats but with different intensities (see Figure 2-all rats, contextual and cued). We also supposed that the variety of cholinergic reactions was revealed by the phenotypical variety of the outbred rats and it was useful for understanding some of the principles of the organization of different forms of cognition.

### 4.2. Cholinergic Composition of the Forms of Cognition in the Normal and 2VO Conditions

The second purpose of our research was a comparative analysis of the behavioural and biochemical parameters for identification of the cholinergic composition of the investigated cognitive functions under normal and 2VO conditions. In the first place it is necessary to note that the results of this research confirmed and expanded the knowledge about cholinergic mechanisms of cognitive functions under normal brain conditions. Our data showed the active involvement of cholinergic projective systems and also regional ones of the cortex and the hippocampus in cognitive processes. Cholinergic synaptic connections with the investigated cognitive functions revealed under normal conditions indicate that each form of cognition has an individual cholinergic synaptic and probably receptor compositions. This conclusion is conformed to the results of investigations, obtained in the Morris water maze and some other behavioural models [34–37].

Then, the data showed the participation of the cholinergic systems not only in mechanisms of learning and working memory, which was repeatedly observed in previous studies [3–5, 45, 61], but also in mechanisms of the inherited abilities and long-term memory. 

Our “inherited abilities” in the contextual task in the Morris water maze was firstly detected by R G Morris and U Frey as a distinct type of memory and termed as “rapid one-trial memory” [62, 63]. We suppose that this function can be inherited. It seems the problem of future discussions. Our data, concerning individual cholinergic organisation of function support contextual inherited abilities as a distinct form of cognition. Morris and Frey observed that allocentric spatial learning can sometimes occur in one trial. Our data concerning the same cholinergic structures associated with inherited abilities 1s2 and long-term memory 3s1 also testify to possible tight interaction between these two forms of cognition.

The involvement of cholinergic projective systems in mechanisms of the long-term memory is usually denied [64–67], and was only discussed in a few studies [36, 68, 69]. Our data confirmed that synaptic populations of cholinergic projective neurons and of the interneurons of the cortex and the hippocampus can have positive and negative connections with cognitive functions. A negative dependence of cognitive functions on cholinergic cortical efficiency was also revealed earlier in cats using a similar methodology for researching the cholinergic synaptic organization of cognitive functions [29]. Therefore, our data demonstrate that the cholinergic mechanisms of learning and memory are more complex than currently perceived. It is evident that this can complicate the detection of cholinergic effects on some cognitive functions by means of nonselective influences on cholinergic efficiency. For example, according to our data in the contextual model, the nonselective pharmacological cholinergic means as well as use of different methods of degeneration of the cholinergic projective systems would certainly have revealed the participation of the cholinergic projective systems in learning 2s2–4 and 3s2–4, but it would probably have concealed a cholinergic participation in the mechanisms of the inherited abilities, the long-term and the working memory. Such results would correspond to the data in the literature [62, 63, 68, 70]. 

From the numerous data in the literature, preservation of the cholinergic projective systems is critical for the success of cognitive processes, and it was thought that cholinergic dysfunction or degeneration results in the impairment of memory in neurodegenerative diseases of different aetiologies. Therefore, we analyzed the connections between reorganization of the cholinergic synaptic pool and impairment of learning and memory under 2VO conditions. The comparative analysis showed that the connections between the functional and cholinergic parameters revealed under normal conditions were practically lost in the ischemic rats. In our research, only impairment of cued long-term memory 2s1 was really dependent on degeneration of the key synaptic population of the cholinergic cortical interneurons, and also, probably, the intact cued memory 3s1 by the unchanged key synaptic population of the cholinergic cortical projective neurons. At the same time, our data also showed different cholinergic compositions of the cognitive functions under 2VO and under normal conditions in as the spatial contextual and the spatial cued models. Under 2VO conditions, most connections of the investigated functions with cholinergic synaptic populations revealed under normal conditions disappeared and new connections with other cholinergic synaptic populations arose. The quantity of cholinergic synaptic populations, involving in mechanisms of the investigated cognitive functions, was considerable reduced. Furthermore, cholinergic connections in general disappeared from the mechanisms of the following forms of cognition: inherited abilities 1s2, learning 2s2–4 and long-term memory 3s1 in the contextual model, and long-term memory 2s1 and 3s1 in the cued model. Moreover, brain region specializations of both the contextual and the cued functions were changed. Cortical cholinergic influences had been completely removed from the contextual functions and hippocampal ones from the cued functions. All considerable differences between cholinergic organisation of the cognitive functions in the normal and 2VO conditions stated above are clear demonstrated in Figure 3. It is important that a consistency between the performances of cognitive functions and their new key cholinergic synaptic populations was found in the majority of the remaining cholinergic-dependent functions under 2VO conditions (from four to six functions). 

Thus, according to our data, we suggest that the normal cholinergic synaptic connections in learning and memory were progressively reduced and changed during chronic ischaemia. It seems that any neurodegenerative pathology undergoes the same processes. It is known that anticholinesterase drugs are only effective in the early stages of Alzheimer's disease (early and mild Alzheimer's disease). It can be noted that the new cholinergic connections with the cognitive functions were not necessarily a consequence of degeneration or dysfunctions in the key cholinergic synaptic populations (it was evident for contextual learning in 3s2–4 and long-term memory in 2s1, that is, these new links could arise by other, indirect reasons). The dependence on the inclusion of cholinergic links in the realization of cognitive functions from the functional background of neuronal environments was recently revealed [64]. This corresponds with the theory by D. A. Sakharov about the nonsynaptic transfer of chemical information [71, 72]. According to this theory, any change in any functional system results in a change in all systems as a result of the change in neuroactive compounds of intercellular environments (the matrix). The changes in the matrix determine the activation of one or another neuronal ensemble which finally determines the behavioural act. From all of these viewpoints, it seems that the main value for cognitive functions is its receptor composition and its change in neurodegenerative pathology. Our data concerning the different consequences on the learning performance under normal and 2VO conditions by the action of RJR and mecamilamine on the same subtypes of nAChR testify to this version.

## 5. Conclusions

It seems that the reasons for changes in the cholinergic organization of cognitive functions in an ischaemic pathology can be any neurodegenerative or, on the contrary, reparative process (sprouting) of cholinergic and noncholinergic synaptic populations. In spite of the brain reparative potentials, the cholinergic and the whole neurochemical organization of cognitive functions under the chronic actions of pathological factors will be formed as optimally as possible under the new conditions. Pathological conditions essentially differ from natural ones. A new organization of cognitive functions will be constructed on neuronal elements which are stable against pathological influences. This new organization can provide an optimum realization of some cognitive functions but not of others. In any case, the study of new key neurochemical links in the organization of cognitive functions may be promising. The plasticity of neurochemical links in the individual organization of certain types of cognition can be used in the future for alternative corrections of vascular and other degenerative dementia.

## Figures and Tables

**Table:** (a)

		I, Contextual		II, Cued		II/I%	*P*
ChAT Activity		mean	SEM	mean	SEM		
CL	sm	1.16	0.18	0.91	0.12	78%	>.05
	Sp	5.57	0.36	5.97	0.46	107%	>.05

CH	sm	0.054	0.008	0.048	0.004	**89%**	<.05
	Sp	0.367	0.037	0.352	0.021	96%	>.05

HL	sm	0.395	0.024	0.320	0.013	**81%**	<.05
	Sp	4.34	0.129	4.21	0.129	97%	>.05

HH	sm	0.022	0.004	0.017	0.003	77%	>.05
	Sp	0.410	0.038	0.415	0.040	101%	>.05

**Table:** (b)

		I, Contextual		II, Cued		II/I%	*P*
Protein content		mean	SEM	mean	SEM		
CL	sm	5.69	0.55	5.00	0.45	88%	>.05
	Sp	4.01	0.14	4.34	0.11	108%	>.05

CH	sm	0.629	0.054	0.677	0.086	108%	>.05
	Sp	2.150	0.054	2.13	0.055	99%	>.05

HL	sm	7.10	0.304	7.49	0.304	105%	>.05
	Sp	2.90	0.195	2.95	0.139	102%	>.05

HH	sm	3.07	0.432	3.45	0.214	112%	>.05
	Sp	2.72	0.104	2.62	0.077	96%	>.05

**Table 2:** *r*-criterion values by the Pearson's test between behavioural performance and ChAT activity in rats in the Morris water maze in the spatial contextual (Contextual) and spatial cued (Cued) behavioural models in the control (Sham) and ischaemic (2VO) conditions. 1s2/1s1, 1s3-4/1s2–4, 2s2–4, 3s2–4, 2s1, 3s1, the abbreviations of the forms of cognition are the same as in Figure 1; sm and Sp, respectively subfractions of the synaptic membranes and the synaptoplasm of the cortical light (CL) and heavy synaptosomes (CH) and hippocampal light (HL) and eavy synaptosomes (HH) as in Table 1. The signs of correlations were placed as plus and minus for the purpose of direct reflection of correlations between performance of the cognitive functions and ChAT activity in the subfractions. The positive (plus) sign of value reflects the positive influence on cognitive function of the corresponding synaptic population and the negative (minus) sign of value reflects accordingly the negative influence on the function of the corresponding synaptic population. In the control and 2VO spatial contextual biochemical rat subgroups *n* = 5 per group; in the control and 2VO spatial cued biochemical rat subgroups *n* = 4 per group. *,**, ***, significant *r*-criterion values of correlations between behavioural performance and ChAT activity (*P* < .05, *P* < .01, *P* < .001, resp.) by the Pearson's test. Significant *r*-criterion values were marked in bold values.

		Contextual						Cued					
sham		1s2	1s3-4	2s2–4	3s2–4	2s1	3s1	1s1	1s2–4	2s2–4	3s2–4	2s1	3s1
CL	Sm	+0.487	−0.359	+0.994***	+1***	+0.295	+0.575	+0.405	− 1***	−0.196	+0.409	−0.234	−0.808
	Sp	+0.961*	−0.242	−0.729	−0.396	+0.689	− 1***	+0.673	+0.515	+0.425	−0.429	+0.249	+0.966*
CH	sm	+0.776	−0.268	−0.441	−0.246	+0.980*	−0.596	+0.617	− 0.982*	−0.617	−0.023	−0.501	−0.503
	Sp	+0.388	−0.343	+0.145	+0.145	+0.965*	−0.219	−0.041	−0.052	+0.958*	+0.997**	+0.978*	−0.369
HL	sm	+0.106	+0.997***	−0.292	−0.263	− 0.967*	+0.207	−0.187	+0.667	+0.759	+0.475	+0.993**	+0.340
	Sp	−0.614	− 0.988*	+0.645	+0.591	+0.460	+0.317	−0.276	−0.249	+0.974*	+1***	+0.658	_0.640
HH	sm	− 0.967*	−0.288	+0.985*	+0.998***	−0.120	+0.979*	−0.413	+0.971*	+0.684	+0.202	+0.730	+0.501
	Sp	− 0.992***	+0.079	+0.601	+0.549	−0.720	+0.993***	+0.644	−0.565	+0.127	+0.689	+0.466	−0.448

2VO		1s2	1s3-4	2s2–4	3s2–4	2s1	3s1	1s1	1s2–4	2s2–4	3s2–4	2s1	3s1

CL	sm	−0.464	−0.270	+0.548	+0.006	+0.242	+0.450	+0.400	+0.321	− 0.981*	+0.986*	−0.677	−0.139
	Sp	−0.656	−0.153	−0.326	−0.797	+0.198	−0.510	+0.577	+0.047	− 0.954*	+0.998**	−0.752	−0.377
CH	sm	+0.082	+0.477	−0.403	−0.750	−0.482	−0.110	−0.307	+0.955*	− 0.995**	+0.403	−0.784	+0.553
	Sp	−0.328	−0.004	−0.231	−0.350	−0.022	+0.304	+0.356	+0.373	− 0.982*	+0.978*	−0.655	−0.095
HL	sm	−0.162	−0.518	−0.302	−0.387	+0.426	+0.375	+0.160	+0.419	−0.782	+0.637	−0.843	+0.133
	Sp	−0.765	− 0.928*	+0.169	−0.119	− 0.908*	+0.145	+0.264	+0.319	−0.756	+0.691	−0.727	+0.027
HH	sm	−0.709	−0.434	−0.454	− 0.905*	+0.448	−0.411	+0.711	−0.346	−0.559	+0.779	−0.634	−0.606
	Sp	−0.497	−0.408	−0.382	−0.753	+0.372	−0.027	−0.604	+0.780	−0.632	+0.050	−0.558	+0.680
